# Signaling pathways and advances in targeted therapy for adenomyosis

**DOI:** 10.3389/fcell.2025.1685525

**Published:** 2025-11-06

**Authors:** Yue Li, Hongyu Zhang, Yaoyao Ding, Yiling Zhao, Yufeng Li, Tiansheng Qin

**Affiliations:** 1 The Second Hospital and Clinical Medical School, Lanzhou University, Lanzhou, China; 2 Department of Gynecology, The Second Hospital and Clinical Medical School, Lanzhou University, Lanzhou, China; 3 Gansu Health Vocational College, Lanzhou, China

**Keywords:** adenomyosis, signaling pathways, targeted therapy, therapeutic advances, pathogenesis

## Abstract

Adenomyosis is a common estrogen-dependent disease, characterized by the invasion of endometrial glands and stroma into the myometrium. It often results in dysmenorrhea, menorrhagia, and infertility, significantly impacting patients’ quality of life. Currently, the etiology and pathogenesis of adenomyosis remain unclear, and existing treatments have limitations. Therefore, further research on the mechanism and treatment of adenomyosis is urgently needed. Studies indicate that adenomyosis involves dysregulation of multiple signaling pathways, including VEGF, Wnt, PI3K, MAPK, NF-κB, cGAS-STING, TGF-β, Hedgehog, and Hippo pathways, which regulate processes such as estrogen and progesterone imbalance, angiogenesis, proliferation and invasion, and the processes of inflammation and fibrosis. This review summarizes the relevant signaling pathways involved in adenomyosis and discusses recent progress in targeted pathway therapies. Additionally, emerging therapeutic strategies such as multi-target combination therapy, epigenetic regulation, and natural products are emphasized as viable avenues for adenomyosis treatment in the future.

## 1 Introduction

Adenomyosis is a benign disease caused by the invasion and proliferation of endometrial glands and stroma into the myometrium. It frequently coexists with endometriosis and/or uterine fibroids, impacting patients’ physical and mental health, fertility, and quality of life. Dysmenorrhea, menorrhagia, secondary anemia, infertility, miscarriage, chronic pelvic pain, and dyspareunia are the main clinical manifestations, and diffuse uterine enlargement is frequently present as well.

As of right now, the pathophysiology and etiology of adenomyosis have not been clearly defined. The widely accepted mechanisms within the field are “invagination” and “metaplasia” (Chen et al., 2024). Recent experiments on humans and animals have validated the theory that endometrial cells invade the myometrium. It is generally accepted that endometrial tissue invades the myometrium through the damaged endometrial-myometrial interface (EMI) and activates tissue damage and repair mechanisms (Margherita et al., 2024). Such damage can result from multiple pregnancies, artificial abortions, and chronic endometritis. Additionally, there are theories involving residual Müllerian ducts and stem cell metaplasia, inflammatory stimulation, epithelial-mesenchymal transition (EMT), and immunological factors. Fortunately, with advancements in next-generation sequencing technology, the mystery of adenomyosis is gradually being uncovered.

Overall, the incidence rate of adenomyosis is 1.03%; when classified by age group, the 41–45 years old group is the high-risk period for the disease, with an annual incidence rate reaching 1.5% (Yu et al., 2020); among patients who have undergone hysterectomy, due to differences in diagnostic criteria, the incidence rate of adenomyosis ranges from 8.8% to 61.5% (Kho et al., 2021). In addition, regarding the treatment of adenomyosis, due to the complexity of adenomyosis and the insufficient emphasis placed on this disease, there are currently no approved therapies for adenomyosis, and related research lags behind that of other gynecological diseases. At present, the treatment for adenomyosis patients is mostly focused on symptomatic treatment. Since the disease is estrogen-dependent, the current primary strategies are to regulate hormone levels to alleviate pain and employ non-steroidal anti-inflammatory medications to reduce inflammation (Kho et al., 2021). When hormonal therapy is ineffective, hysterectomy is often used. For adenomyosis patients with fertility needs, hysterectomy, as a traditional radical treatment option, has significant limitations. This treatment method directly leads to the loss of fertility, which is fundamentally contradictory to the core treatment goal of such patients (Bulun et al., 2023). Furthermore, the diagnosis of adenomyosis also relies on hysterectomy, and due to differences in the definition of adenomyosis between the histopathology and the imaging point of view, the diagnosis of adenomyosis also faces challenges. Therefore, it is crucial to further develop targeted therapeutics for adenomyosis. Multiple studies have confirmed that the pathogenesis of adenomyosis is related to several signaling pathways, such as VEGF,Wnt, PI3K, MAPK, NF-κB, cGAS-STING, TGF-β, Hedgehog, and Hippo pathways. These signaling pathways are involved in various mechanisms of adenomyosis and crosstalk with each other, relating to pathological features such as hormonal imbalance, angiogenesis, proliferation and invasion, and the processes of inflammation and fibrosis. Additionally, a series of related studies have also found the potential of targeted signaling pathway therapy in adenomyosis. Therefore, the signaling pathways linked to adenomyosis and the corresponding targeted therapies will be the main focus of this review.

## 2 Overview of signaling pathways in adenomyosis

### 2.1 Hormone-related signaling pathways

Adenomyosis is characterized by excessive estrogen expression and progesterone resistance. Increased estrogen levels promote proliferation of ectopic endometrial tissue, while progesterone plays a crucial role in inhibiting estrogen-induced proliferation. The imbalance between estrogen and progesterone in adenomyosis will jointly facilitates the proliferation of ectopic lesions (Dias Da Silva et al., 2024).

ESR1 and ESR2 encode ERα and ERβ respectively, which are key targets of estrogen (E2) in the endometrium. In adenomyosis, decreased expression of ESR1 and increased expression of ESR2 can be observed (Bulun et al., 2023), attributable to the methylation-mediated suppression of ESR2 in normal endometrial tissue. Conversely, in ectopic endometrial tissue, ESR2 expression is regulated by an unmethylated, active promoter region. Additionally, the ESR2 protein interacts with the ESR1 gene promoter, inhibiting ESR1 activity. Consequently, ESR1 is suppressed, while ESR2 is overexpressed in proliferative ectopic endometrial tissue. In addition, estrogen can also increase the transcription of VEGF through ERα (Cignarella et al., 2022), promoting angiogenesis, which plays a critical role in adenomyosis. Excessive estrogen expression thus contributes to lesion proliferation and angiogenesis, making the regulation of estrogen levels a vital aspect of adenomyosis treatment.

In addition to abnormal estrogen levels, adenomyosis is characterized by progesterone resistance, which disrupts the immune microenvironment. This resistance is primarily due to decreased expression of the progesterone receptor PGR-B, which is mainly resulting from DNA methylation-mediated suppression of PGR-B in the stromal cells of the adenomyosis. Conversely, membrane progesterone receptors (mPRs) or progesterone receptor membrane components (PGRMCs) are highly expressed in adenomyosis, indicating the complex regulation of progesterone in this disease (Sztachelska et al., 2022). During this intricate process, progesterone also mediates prolactin (PRL) secretion, which has been shown in studies to possess anti-apoptotic effects and support tumor growth in certain types. In animal models of adenomyosis, PRL and its receptor are highly expressed within the lesions (Sztachelska et al., 2022). Furthermore, progesterone (P4) influences inflammation-related signaling pathways, such as inhibiting the NF-κB pathway; however, in the context of progesterone resistance, the inhibitory effect on this signaling pathway is reduced, promoting the development of ectopic lesions (Vannuccini et al., 2022).

As can be seen from the above, adenomyosis is significantly influenced by the intricate process of estrogen and progesterone balance. Estrogen and progesterone are regulated by various factors, and a slight change in this process may disrupt the balance. Moreover, this balance plays a key role in adenomyosis. For this reason, further investigation into the regulatory mechanisms of estrogen- and progesterone-related signaling pathways is of significant importance for the development of targeted therapies for adenomyosis.

### 2.2 Angiogenesis signaling pathways

#### 2.2.1 VEGF/HIF

The primary symptom of adenomyosis is abnormal uterine bleeding. Studies of hysterectomy specimens have revealed increased expression of angiogenic factors and a higher vascular density (Harmsen et al., 2025). Angiogenesis refers to the formation of new capillaries from existing blood vessels during physiological and pathological processes. The VEGF family and their receptors are key molecules in angiogenesis. Among them, VEGFA exerts its effects by binding to VEGFR1 and VEGFR2, inducing the formation of both physiological and pathological blood vessels (Pérez-Gutiérrez and Ferrara, 2023). In addition, hypoxia is particularly important in the process of angiogenesis, and HIF-1 is essential in hypoxia. HIF-1 induces the activation of many downstream genes, including VEGF, by interacting with the hypoxia response element (HRE), thereby stimulating angiogenesis (Meo and de Nigris, 2024). Research has demonstrated that HIF-1 is highly expressed in adenomyosis (Guo et al., 2021), which can enhance VEGF-induced angiogenesis. Furthermore, the ectopic endometrium in adenomyosis needs to complete ectopic colonization through angiogenesis, so inhibiting angiogenesis is expected to become a promising therapeutic approach for adenomyosis.

### 2.3 Cell proliferation and invasion signaling pathways

#### 2.3.1 Wnt

Adenomyosis is closely associated with the abnormal activation of the Wnt signaling pathway. Previous studies have demonstrated that nuclear translocation of β-catenin is detected in adenomyosis; in other words, β-catenin is overexpressed in adenomyosis (Feng et al., 2017). This is because when Wnt binds to FZD and LRP5/6 receptors, the pathway is activated, recruiting cytosolic protein Dishevelled (DVL) to bind FZD, promoting the multimerization of the destruction complex, and recruiting its components to the plasma membrane. This leads to the inhibition of β-catenin degradation, allowing β-catenin to enter the nucleus and bind to TCF/LEF transcription factors to regulate gene expression (Maurice and Angers, 2025). The Wnt/β-catenin pathway is essential for normal uterine physiological functions; dysregulation of this signaling pathway can lead to pathological states, affecting cellular proliferation and survival, and promoting lesion invasion and migration through EMT (Yoo et al., 2020; Li et al., 2025). Furthermore, emerging research has shown that the level of ARG2 is increased in adenomyosis, and ARG2 plays a role in adenomyosis by regulating the Wnt/β-catenin signaling pathway (Xu et al., 2024). This further reveals the role of this signaling pathway in adenomyosis and also provides a new target for the treatment of adenomyosis.

#### 2.3.2 PI3K

Although adenomyosis is a benign disease, its proliferation and invasion capabilities are very similar to the biological behaviors of tumors. The phosphatidylinositol 3-kinase (PI3K) signaling pathway is of great significance in the activation of tumors and has been confirmed to be activated in rat models of adenomyosis (Yu et al., 2022; Yang et al., 2025). Activated PI3K catalyzes the conversion of PIP2 to PIP3. As a second messenger, PIP3 activates the downstream signaling protein AKT. The activated Akt relieves the inhibition of mTORC1 by phosphorylating the TSC complex, thereby regulating cell growth and metabolism (Yu et al., 2022). Furthermore, compared to the control group without endometrial lesions, patients with adenomyosis exhibit upregulated expression of PI3K and Akt genes and proteins, thereby promoting the proliferation of endometrial cells (Zhang et al., 2022). Therefore, targeting the PI3K signaling pathway is expected to become a therapeutic direction for controlling the proliferation and progression of adenomyosis lesions.

#### 2.3.3 MAPK

The proliferation of adenomyosis is associated with the MAPK signaling pathway and genetic variations, among which the activating mutation of KRAS is the most common genetic variation in adenomyosis epithelial cells (Bulun et al., 2023). Mutations in KRAS can activate the downstream mitogen-activated protein kinase (MAPK) signaling pathway, leading to cell proliferation and differentiation (Bulun et al., 2021). In addition, studies have found that co-dysregulated circRNAs exist in the eutopic endometrium and EMI of adenomyosis, and they induce endometrial invagination through the MAPK signaling pathway, promoting the progression of adenomyosis (Guo et al., 2022). Based on its downstream components, the MAPK signaling pathway can be roughly divided into three categories, including ERK, JNK, and p38 (Anjum et al., 2022). Among them, the classical RAF-MEK-ERK pathway is activated by receptor tyrosine kinases. After RAS binds to GTP, it activates RAF. RAF phosphorylates and activates MEK1/2, and then MEK further phosphorylates ERK to activate it, which is involved in cell proliferation, survival and differentiation (Lavoie et al., 2020). The p38 MAPK signaling pathway mainly mediates inflammation and decidualization of endometrial stromal cells (Tong et al., 2022), while JNK mainly mediates the regulation of cell apoptosis (Anjum et al., 2022). Therefore, the MAPK signaling pathway plays an important role in the proliferation and differentiation of adenomyosis.

### 2.4 Inflammation and fibrosis signaling pathways

#### 2.4.1 NF-κB

The NF-κB signaling pathway plays a crucial role in the inflammatory response in adenomyosis. In addition to being closely related to the inflammatory response, the NF-κB pathway also regulates the expression of genes related to cell proliferation, survival, migration, and drug resistance (Kobayashi, 2023b). In adenomyosis, the NF-κB pathway is in a state of abnormal activation, which is mainly caused by factors such as TNF-α, IL-1β, LPS, etc. Subsequently, IKKα and IKKβ phosphorylate IκBα, leading to its ubiquitination and proteasome-mediated degradation, thereby releasing p50/RelA as a transcription factor to activate the transcription of target genes (Guo et al., 2024). Studies have shown that the levels of P50 and P65 proteins are significantly elevated in the adenomyosis group compared to the control group (Li et al., 2013), further confirming the abnormal activation of this pathway in adenomyosis. This series of processes leads to inflammation, extracellular matrix disassembly, and abnormal cell proliferation in the endometrium of women with adenomyosis (Zhai et al., 2022), affecting the pathogenesis and progression of adenomyosis.

#### 2.4.2 cGAS-STING

The cGAS-STING pathway is an essential signaling mechanism in innate immunity, and it plays an important role in chronic inflammation and abnormal cell proliferation of adenomyosis (Wang et al., 2024). Studies have demonstrated that in the endometrial tissues of patients with adenomyosis, the cGAS, STING, interferon-α (IFN-α), IFN-β, TANK-binding kinase 1 (TBK-1), and tumor necrosis factor-α (TNF-α) mRNA and protein levels are all increased (Lin et al., 2021), which suggests the abnormal activation of this signaling pathway in adenomyosis. The cGAS/STING signaling pathway consists of the cytoplasmic DNA sensor cGAS and the transmembrane adapter protein STING. cGAS binds to double-stranded DNA (dsDNA) to form the second messenger cyclic GMP-AMP (cGAMP), after which cGAMP activates STING proteins on the endoplasmic reticulum to oligomerize, translocate to the Golgi apparatus, and be modified by palmitoylation. This modification facilitates the recruitment of TBK1 and IRF3, inducing the expression of pro-inflammatory cytokines and type I interferons, and activating the NF-κB pathway at the same time (Dvorkin et al., 2024). Therefore, targeting the cGAS-STING signaling pathway may be a potential therapeutic direction for addressing the chronic inflammatory response in adenomyosis.

#### 2.4.3 TGF-β

Fibrosis and the EMT caused by TGF-β are important processes in adenomyosis (Kay et al., 2020; Yoo et al., 2020). The classical TGF-β/Smad signaling pathway is initiated when TGF-β binds to TGF-β receptor type 2 (TGFR 2) and recruits TGFR 1 to form a receptor complex, after which the phosphorylated TGFR 1 activates Smad 2 and Smad 3. Once activated, Smad 4 is recruited and binds to Smad 2/3, and the complex translocates into the nucleus for transcription (Peng et al., 2022). In adenomyosis, fibrosis is an important pathological feature. Abnormally activated TGF-β signaling pathway can promote the formation of fibrosis. Among them, the TGF-β signaling pathway can directly act on fibroblasts to promote the accumulation of extracellular matrix (ECM), such as collagen and αSMA, thereby facilitating the formation of fibrosis (Kobayashi et al., 2020). It can be seen that inhibiting the TGF-β signaling pathway will be a highly promising research direction in the treatment of adenomyosis.

### 2.5 Emerging signaling pathways

#### 2.5.1 Hedgehog

The process of organismal growth and development is significantly influenced by the Hedgehog signaling pathway. Among them, Indian Hedgehog (IHH) is involved in tumorigenesis and embryonic development. In the invaginated microenvironment of adenomyosis, SFRP5+ epithelial cells can cause angiogenesis and endometrial proliferation through autocrine and paracrine IHH, promoting disease progression (Li et al., 2025). In the presence of HH ligands, the ligands bind to Patched (PTCH), relieving the inhibitory effect on Smoothened (SMO), thereby activating the downstream signaling pathway. The GLI family of transcription factors are downstream effector molecules, and their activity state determines the transcription of target genes (Song et al., 2022). Previous studies have also found that IHH, its transcription factor SOX 17, receptor PTCH 1, and target gene HHIP are all highly expressed in adenomyosis (Li et al., 2025). The abnormal expression of this series of molecules fully indicates that the Hedgehog signaling pathway plays an important role in adenomyosis.

#### 2.5.2 Hippo

The Hippo signaling pathway plays an important role in cell proliferation, apoptosis, tissue metabolism, and organ formation. It has been confirmed that the Hippo signaling pathway is in an inactivated state in adenomyosis, while activating this pathway can reverse EMT and reduce cell proliferation and invasion (Jin et al., 2023). The core components of this signaling pathway include Mst1/2, Lats1/2, YAP, and TAZ, among which YAP is a key protein of the Hippo signaling pathway. When the Hippo signaling pathway is turned off, YAP/TAZ is in a dephosphorylated state, and binds to transcription factors such as TEAD to activate transcription, leading to cell proliferation. When the pathway is turned on, YAP/TAZ is phosphorylated, binds to 14-3-3 protein and then is degraded, thereby inhibiting transcription (Li and Guan, 2022). Therefore, the Hippo signaling pathway regulates transcription through YAP/TAZ, and the inactivation of the Hippo pathway and the dysregulation of related molecules provide new directions in the research and treatment of adenomyosis.

**FIGURE 1:**
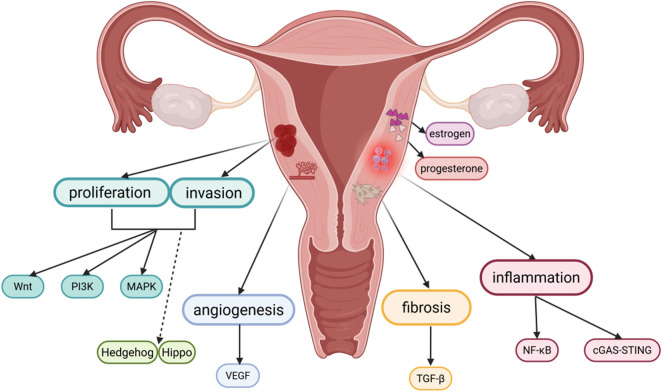
Pathophysiology of adenomyosis and related signaling pathways. Created in BioRender. yue, L. (2025) https://BioRender.com/crjj5xm.

**FIGURE 2:**
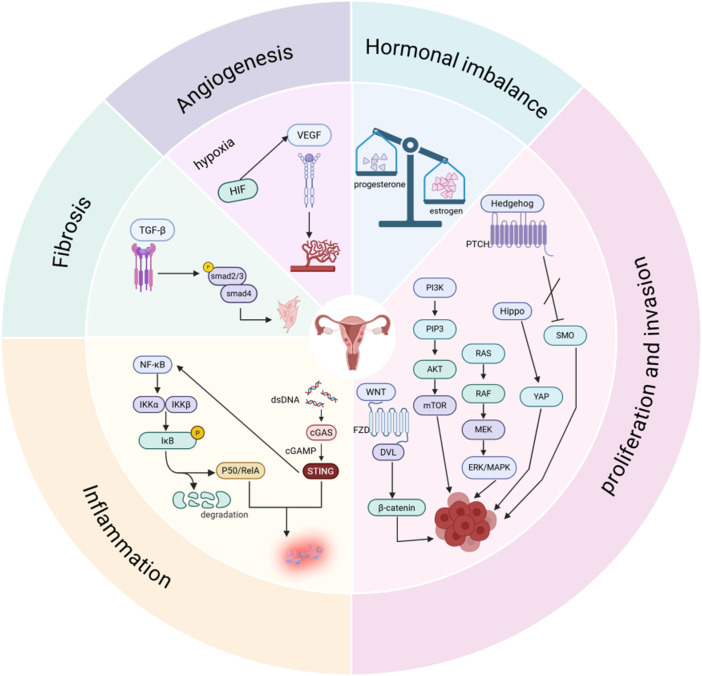
Signal pathways involved in the pathological process of adenomyosis. Created in BioRender. yue, L. (2025) https://BioRender.com/ugx44su.

A brief overview of the signaling pathways involved in adenomyosis reveals that the abnormal activation of these pathways plays a significant role in the pathogenesis and progression of adeno-myosis. Hormonal balance, angiogenesis, cell invasion and proliferation, and the processes of inflammation and fibrosis are all regulated by this network of interconnected signaling pathways (Figures 1, 2). Currently, there are still many limitations in the treatment of adenomyosis. The mechanisms of action of these signaling pathways in adenomyosis must be more thoroughly understood in order to develop the treatment of adenomyosis. Based on current understanding, targeted therapies against specific signaling pathways have been applied in oncology with notable efficacy. These studies also provide new potential targets for the treatment of adenomyosis. The current state of targeted signaling pathway treatments for adenomyosis is covered in the following.

## 3 Advances in targeted signal pathway therapies for adenomyosis

### 3.1 Estrogen and progestogen-related therapies

Among progestogen medications, the first-line treatment for alleviating symptoms such as heavy menstrual bleeding and dysmenorrhea in adenomyosis is the levonorgestrel-releasing intrauterine system (LNG-IUS). The LNG-IUS exerts its effects by acting on the signaling pathway mediated by progesterone receptors and can also achieve a contraceptive effect (Kho et al., 2021). Currently, emerging selective progesterone receptor modulators (SPRMs) can modulate progesterone receptor activity and influence the transcription of target genes. For example, the representative drug ulipristal can significantly reduce dysmenorrhea and menstrual blood loss; however, post-marketing reports have indicated cases of hepatic failure, leading to restrictions on its use (Yoon and Yuk, 2021). Another SPRM, mifepristone, which has been proven to be effective and safe in the treatment of adenomyosis, has been shown in studies to affect the expression of caspase 3 in adenomyosis tissues and induce cell apoptosis through the mitochondria-dependent signaling pathway (Che et al., 2020). At present, due to the limited data evaluating the role of SPRMs, the treatment with SPRMs remains controversial, and further research on the efficacy and safety of SPRMs is needed in the future. In addition, SIRT1, as a class III histone deacetylase, inhibits progesterone receptors, leading to progesterone resistance. SIRT1 also affects endometrial receptivity by downregulating FOXO1, contributing to infertility, so SIRT1 is a promising therapeutic target. Current research on SIRT1 inhibitors, such as EX-527, demonstrates their potential to reduce ectopic lesion proliferation (Marquardt et al., 2023).

Furthermore, when progestogen therapy is intolerable or has limited therapeutic effects, gonadotropin-releasing hormone (GnRH) can be an alternative. GnRH acts on the hypothalamic-pituitary-ovarian (HPO) axis. Long-term use of GnRH leads to desensitization of pituitary receptors, thereby inhibiting the secretion of follicle-stimulating hormone (FSH) and luteinizing hormone (LH), and ultimately reducing estrogen production (Etrusco et al., 2023). A study found that in patients with severe adenomyosis, after treatment with GnRH agonists to inhibit pituitary function, the estrogen level remained high; however, the therapeutic effect was significantly improved when combined with aromatase inhibitors (Humaidan et al., 2023). This indicates that the combined use of GnRH agonists and aromatase inhibitors can achieve a better effect in inhibiting estrogen. The activity of aromatase is regulated through multiple signaling pathways such as PI3K and MAPK, while aromatase inhibitors reduce estrogen synthesis by inhibiting the activity of aromatase (Liu et al., 2021b). Nevertheless, there have been previous reports of two cases where patients treated with aromatase inhibitors developed endometrial cancer (Yanase et al., 2017). Currently, there is a lack of extensive research to confirm this, and further verification of the safety of aromatase inhibitors is necessary.

### 3.2 Anti-angiogenesis

Increased angiogenesis leads to a significant elevation in the mean vascular density (MVD) of the endometrium, and the new blood vessels have high fragility and permeability, thereby contributing to abnormal uterine bleeding in adenomyosis (Harmsen et al., 2019). Inhibiting angiogenesis has long been a widely used target in tumor treatment (Harmsen et al., 2025). With the in-depth research on angiogenesis, this target has also been applied to the treatment of benign diseases, such as rheumatoid arthritis, age-related macular degeneration, and other diseases related to abnormal angiogenesis (Pérez-Gutiérrez and Ferrara, 2023).

The increased expression of angiogenesis markers in adenomyosis provides possibilities for its treatment. In a mouse model of tamoxifen-induced adenomyosis, axitinib, a VEGF-R2 tyrosine kinase inhibitor, can inhibit angiogenesis and reduce the severity of adenomyosis (Harmsen et al., 2025). In addition, platelet activation is also related to angiogenesis because it occurs together with the release of TGF-β1, leading to EMT and the transdifferentiation of fibroblasts into myofibroblasts. Although antiplatelet therapy for adenomyosis by inhibiting angiogenesis seems to be a promising therapeutic direction, long-term application of antiplatelet therapy has the risk of bleeding and requires more cautious use (Harmsen et al., 2025). It should be noted that since angiogenesis is very important in the normal operation of the menstrual cycle, anti-angiogenic therapy needs to be selective to avoid affecting the normal physiological functions (Harmsen et al., 2025). For adenomyosis patients who want to preserve their fertility, selective inhibition of angiogenesis may represent a novel therapeutic approach, and this treatment method provides the possibility of preserving fertility (eBioMedicine, 2025).

### 3.3 Anti-cell proliferation and invasion

According to current research, targeting the Wnt signaling pathway can be carried out from three aspects: targeting Wnt production, stabilizing the destruction complex, and using Wnt surrogate molecules. To begin with, PORCN inhibitors such as LGK974 that target Wnt production can block Wnt secretion (Liu et al., 2022). Then, TNKS inhibitors that stabilize the destruction complex can stabilize AXIN1. Finally, Wnt surrogate molecules include bifunctional antibodies, such as scFv-DKK1c and tetravalent antibodies (FLAg), which activate signals through high affinity, as well as next-generation surrogates (NGS) designed by DRPB, which have higher specificity (Maurice and Angers, 2025). Although targeting the Wnt signaling pathway is very attractive in the treatment of adenomyosis, the safety and selectivity of its targeted drugs still need further research and improvement. Since the Wnt signaling pathway plays an important role in normal development and maintaining tissue homeostasis, non-selective systemic application of targeted drugs for the Wnt signaling pathway may have adverse effects on normal tissues (Tufail et al., 2025).

In addition, studies have demonstrated the presence of highly heterogeneous fibroblast-like cells within the uterine tissue of patients with adenomyosis. WNT signal transduction involves the differential expression of secreted frizzled-related proteins (SFRP), which act as decoy receptors for WNT and may play a key role in the pathophysiology of adenomyosis (Yildiz et al., 2023). Previous research indicates that SFRP2 functions as an inhibitor of WNT signaling, capable of downregulating estrogen-dependent β-catenin activity and suppressing cellular proliferation. The loss of SFRP2 expression in the endometrium may lead to epithelial cell growth and invasion. Therefore, SFRP2 could serve as a key predictive biomarker for the proliferative potential of epithelial cells in adenomyosis (Yildiz et al., 2023).

Since the activation of the PI3K signaling pathway in adenomyosis can promote the proliferation of endometrial cells, targeting this pathway thus has anti-proliferative functions. Furthermore, research has also demonstrated that fibrosis of the myometrium in adenomyosis involves dysregulation of the PI3K/AKT signaling pathway, and inhibition of this pathway can alleviate fibrosis in adenomyosis (Yang et al., 2025), which provides an important basis for therapies targeting the PI3K signaling pathway. Currently, various drugs targeting the PI3K pathway have been developed, which can be classified based on their specific molecular targets into mTORC1 inhibitors, pan- PI3K inhibitors, pan-AKT inhibitors, dual PI3K/mTOR inhibitors, and subtype-specific PI3K inhibitors, etc., (Nunnery and Mayer, 2020). Common adverse effects associated with these agents include hyperglycemia, dermatitis and rash, stomatitis, diarrhea, nausea, and fatigue. Several of these drugs can inhibit all subtypes of class IA PI3K, but their broad action is associated with significant toxicity and off-target effects, which will have adverse impacts on normal tissues during the treatment of adenomyosis. Therefore, developing specifically selective targeted drugs and reducing the toxic effects of drugs are particularly important for the safe and effective treatment of adenomyosis. Proteolysis-targeting chimeras (PROTACs) represent a promising approach to address drug toxicity (Kelly et al., 2024), as they selectively induce specific mutations in PI3K while preserving normal PI3K signaling in healthy tissues. Thus, this targeted strategy holds substantial potential for improving both the efficacy and safety of therapeutic interventions.

Currently, research on treatments related to the MAPK signaling pathway mainly focuses on targeting RAF, MEK, or ERK, with most of them in clinical and preclinical studies. However, the clinical application of these drugs also has some limitations, such as the development of drug resistance, toxicity, and abnormal activation of signaling pathways caused by the use of inhibitors (Ullah et al., 2022). DUSP4 can inhibit cell proliferation by dephosphorylating and suppressing the activity of MAPK(Mandal et al., 2023). In addition, the cryo-electron microscopy structure of the BRAF-MEK1- 14-3-3 complex provides insights for the development of novel inhibitors targeting the assembly of active MAPK signaling complexes (Ullah et al., 2022). Regarding the treatment of adenomyosis involving the MAPK signaling pathway, studies have shown that HAND2-AS1 regulates the FGFR-mediated MAPK signaling pathway through HAND2. The activated FGFR promotes the proliferation and migration of endometrial cells, ultimately participating in the pathogenesis and progression of adenomyosis. Therefore, HAND2-AS1 may serve as a novel therapeutic target for adenomyosis (Xiang et al., 2019). Furthermore, the use of a traditional Chinese medicine called Qiu’s Neiyi Recipe can also significantly reduce the increase in inflammatory factors in a mouse model of adenomyosis by inhibiting the MAPK signaling pathway and alleviate the severity of adenomyosis (Ying et al., 2021). However, the role of this recipe in adenomyosis has not been comprehensive, and further research is needed.

### 3.4 Anti-inflammation and anti-fibrosis

Adenomyosis is a chronic inflammatory disease. Various inflammatory cells and factors are activated in adenomyosis. The imbalance of inflammatory status may affect the invasion of endometrial cells, the recruitment and activation of immune cells, as well as their interactions. Persistent inflammation can also affect endometrial receptivity and decidualization, leading to preg-nancy disorders (Kobayashi, 2023a). Therefore, targeting inflammation-related signaling pathways, such as NF-κB and cGAS-STING, may alleviate the symptoms of adenomyosis. The use of inhibitors, natural compounds, and metabolites to modulate these inflammatory pathways has been a major focus of ongoing research. Numerous studies have demonstrated the promising clinical potential of targeting inflammation-associated signaling pathways in the management of this condition.

Currently, drugs targeting the NF-κB signaling pathway mainly include two types of agents that inhibit IκB degradation: IKK inhibitors and proteasome inhibitors. Firstly, IKK inhibitors such as aspirin and sulfasalazine exert therapeutic effects by inhibiting IKKβ to prevent IκB degradation. Secondly, proteasome inhibitors like bortezomib and carfilzomib block IκB degradation by suppressing proteasome activity (Guo et al., 2024). In addition to the above two categories of drugs, NKILA, a long non-coding RNA (lncRNA), can also inhibit IκBα phosphorylation, thereby suppressing NF-κB activation and EMT (Ahmad et al., 2022). Furthermore, monoclonal antibodies such as anti-PD-1/PD-L1 and anti- IL-1 can inhibit pathway activation by blocking ligand-receptor interactions. Other agents include tacrolimus, which inhibits nuclear translocation, and glucocorticoids, which suppress DNA binding (Guo et al., 2024). Interestingly, melatonin (N-acetyl-5-methoxytryptamine), an antioxidant, can block NF- κB signaling to reduce the production of pro-inflammatory cytokines such as TNF-α and IL-1β, and has positive effects on uterine development and endometrial receptivity, indicating its potential as a therapeutic candidate for improving fertility in patients with adenomyosis (Guan et al., 2022). Emerging studies have shown that ARG2 levels are elevated in patients with adenomyosis. ARG2 gene silencing mediates cell apoptosis and arrests cells in the G0/G1 phase by inhibiting the NF-κB and Wnt/β-catenin signaling pathways. These findings suggest that ARG2 plays an important role in adenomyosis, highlighting the potential of targeting ARG2 as a therapeutic strategy for adenomyosis (Xu et al., 2024).

Drugs targeting the cGAS-STING signaling pathway can be broadly classified into two categories: cGAS-targeting agents and STING-targeting agents. Currently, cGAS inhibitors include catalytic site inhibitors and DNA-binding antagonists, while STING-targeting drugs comprise CDN-binding antagonists and STING palmitoylation inhibitors (Zhang et al., 2023). At present, it is still unknown which of the two parts, targeting cGAS or STING, is superior in terms of improving efficacy or safety. Research on adenomyosis have shown that knocking out STING results in decreased expression of IL-6 and IFN-α, along with reduced migration and invasion capabilities of endometrial stromal cells (ESCs) (Wang et al., 2024). This suggests that targeting the cGAS-STING pathway could serve as an effective therapeutic strategy for adenomyosis, providing important experimental evidence for the treatment of adenomyosis by targeting the cGAS-STING signaling pathway. In addition, due to the chronic inflammatory mechanism of adenomyosis and the fact that targeting this signaling pathway can affect the anti-infection ability, it is particularly important to understand the minimum inhibition level for the treatment targeting the cGAS-STING signaling pathway (Decout et al., 2021). If a balance can be found between using the cGAS-STING signaling pathway for treatment and not excessively impairing immune capacity, cGAS-STING could become a very advantageous therapeutic target (Decout et al., 2021).

As a chronic disease, adenomyosis is characterized by fibrosis, which is a key pathological feature. Numerous studies have confirmed that inhibiting TGF-β signaling and its downstream pathways can markedly suppress fibrosis in the disease. Recent preclinical research has demonstrated the efficacy of targeting the TGF-β signaling pathway; however, due to the complexity of fibrosis formation, therapeutic interventions aimed at this pathway have shown limited success (Peng et al., 2022). Fibroblasts serve as the central effector cells in fibrosis and are the primary targets of TGF-β under fibrotic conditions (Frangogiannis, 2020). Given that fibrosis functions as a tissue repair mechanism, simply inhibiting fibrosis may affect the normal repair function of the body. Moreover, adenomyosis is a chronic inflammatory disease requiring long-term administration of TGF-β inhibitors, which increases the risk of impaired wound healing, vascular defects, immune dysregulation, or tumorigenesis (Frangogiannis, 2020). These issues highlight the challenges that need to be addressed in TGF-β-targeted therapies. Previous studies have demonstrated that β-catenin can induce EMT in adenomyosis by activating the TGF-β signaling pathway, characterized by decreased E-cadherin expression and increased vimentin levels (Yoo et al., 2020). Further research has shown that the use of the TGF-β inhibitor pirfenidone can suppress the EMT process and invasiveness of Ishikawa cells following β-catenin activation, as well as reverse the decreased expression of E-cadherin (Yoo et al., 2020). These findings suggest that pirfenidone has potential therapeutic value in treating adenomyosis through the inhibition of the TGF-β signaling pathway. Furthermore, there are some emerging studies on inhibiting TGF-β. It has been reported that circRNA can inhibit the development of colorectal cancer by suppressing the TGF-β/Smad signaling pathway (Zheng et al., 2022). Additionally, studies indicate that circEIF3I expression is elevated in pancreatic cancer, where it promotes SMAD3 recruitment to facilitate TGF-β-induced MMP activation, thereby inhibiting tumor proliferation (Zhao et al., 2023). In summary, these findings illustrate the emerging functions of circRNA in EMT and mediating TGF-β, and they also hold corresponding potential for the treatment of adenomyosis.

### 3.5 Emerging signaling pathway therapies

Since the successful application of Hedgehog pathway inhibitors in the treatment of medulloblastoma, these agents have been extensively used for basal cell carcinoma, with proven efficacy and acceptable tolerability. Currently, three drugs are approved for clinical use: vismodegib, sonidegib, and glasdegib (Zhang and Beachy, 2023). A study on inflammatory bowel disease has confirmed that the Hedgehog signaling pathway is upregulated in inflammatory bowel disease and is associated with the expression of Th17 markers (Hanna et al., 2022). Additionally, in mouse models, the application of the small molecule Hedgehog pathway inhibitor vismodegib has been shown to reduce the degree of inflammation in the disease. Thus, it is speculated that since adenomyosis is a chronic inflammatory disease, it might also have therapeutic effects, but further research is needed to validate this hypothesis. Presently, most Hedgehog pathway inhibitors are SMO small molecule antagonists. A recent study on targeting PTCH1 showed that Fab^6^ᴴ^3^ obtained from anti-Patched-1 monoclonal antibodies can affect the binding of SHH to PTCH1, thereby inhibiting the activation of HH signaling (Hu et al., 2025). Due to the increased expression of PTCH1 in adenomyosis, this targeted therapy can more specifically inhibit the Hedgehog signaling pathway, potentially representing a promising avenue for future research. Furthermore, this pathway is also regulated through primary cilia, and its effect on stem cells provides new translational ideas for regeneration and adenomyosis treatment (Zhang and Beachy, 2023).

In a study, it was found that the expression of let-7a is downregulated in adenomyosis, while YAP1 and TAZ are upregulated. Upregulating let-7a activates the Hippo-YAP1 axis, which inhibits the proliferation and promotes apoptosis of smooth muscle cells in the EMI. Therefore, let-7a and the Hippo-YAP1 axis may serve as novel therapeutic targets for adenomyosis (Huang et al., 2021). In addition, studies have shown that YAP1 can inhibit the expression of progesterone receptors. By using specific YAP1 inhibitors, the expression of progesterone receptors can be increased and progesterone resistance can be improved (Lin et al., 2023). One of the specific YAP inhibitors, Verteporfin, was found in the study to reduce the migration of Ishikawa cells and inhibit the EMT process (Jin et al., 2023). All the above can indicate that targeting the Hippo signaling pathway has potential in the treatment of adenomyosis. However, since Hippo is differentially regulated in different cell populations and different diseases, this will increase the difficulty of further research on targeting the Hippo signaling pathway.

From the above content regarding the treatment related to signaling pathways in adenomyosis, it can be known that the signaling pathways related to adenomyosis are intricate and mutually crosstalk, which poses challenges to the treatment targeting signaling pathways. The activated signaling pathways may vary among different patients, and most adenomyosis patients are accompanied by uterine fibroids or endometriosis, which are not conducive to the application of precise targeted therapy. Fortunately, the majority of medications treat these diseases simultaneously (Etrusco et al., 2023). However, the long-term effectiveness and related safety of drugs need to be confirmed by further studies. Researchers should pay attention to whether long-term use of drugs will damage the function of normal organs and the risk of cancerization. Currently, most therapies targeting signaling pathways are in preclinical stages. More research is required to bridge the gap between preclinical research and clinical practice, as well as move forward from animal models to human applications.

## 4 Emerging research directions and prospects for the treatment of adenomyosis

### 4.1 Multi-target combination therapy

The pathogenesis of adenomyosis is not caused by the activation of a single signaling pathway, but involves the cross-activation of multiple signaling pathways. Therefore, therapies targeting only one pathway may fail to achieve the desired therapeutic effect or may even lead to drug resistance. For example, studies have shown that Wnt signaling crosstalks with pathways such as PI3K/Akt and MAPK, and inhibition of a single target is prone to cause compensatory activation (Tufail et al., 2025). In addition, the mechanism of the MAPK signaling pathway is complex, and it also crosstalks with multiple signaling pathways. Combining drugs that target the MAPK signaling pathway with those that target components of related pathways such as PI3K, AKT, mTOR, or TGF-β, including immune system effectors in the tumor microenvironment, this multi-target combination therapy can not only improve the therapeutic effect of drugs but also avoid the occurrence of drug resistance. Therefore, combination therapy will be the focus of future research (Anjum et al., 2022). At present, there are multi-target drugs, such as melatonin and imperatorin, which are being studied in the treatment of endometriosis. Melatonin can inhibit the NF-κB pathway to exert anti-inflammatory effects, inhibit estrogen-induced cell proliferation, have a direct impact on pain, and also reduce angiogenesis by regulating VEGF. Currently, melatonin has entered the clinical trial stage and is a potential multi-target therapeutic drug (Hung et al., 2021). As for imperatorin, it can inhibit the secretion of TNF-α and IL-6 by suppressing the activation of the PI3K/Akt/NF-κB signaling pathway, thereby inhibiting the progression of the disease (Ma et al., 2021). Although multi-target therapy is a research direction with great potential, the effect on multiple targets at the same time may expand the toxic effects of drugs, so it is necessary to pay extra attention to the adverse reactions of such drugs when researching them.

### 4.2 Epigenetic regulation

Research indicates that a significant number of epigenetic abnormalities are present in the stromal cells of adenomyosis, distinguishing them from the endometrial stromal cells of unaffected individuals. These abnormalities result in dysregulated mRNA and protein expression of genes such as aromatase, progesterone receptors, and estrogen receptors (Bulun et al., 2023).

Research has shown that the total m6A level in the myometrium of adenomyosis is reduced, which is related to the differential expression of the m6A methyltransferase METTL3 and the regulator ZC3H13(Huang and Chen, 2023). Previous studies have found that decreased expression of METTL3 leads to a reduction in m6A modification levels, and downregulation of METTL3 affects the migration and invasion abilities of endometrial cells by inhibiting the maturation of pri-miRNA (Huang and Chen, 2023). ZC3H13, an auxiliary subunit of the m6A methyltransferase complex, when aberrantly expressed, can affect the overall modification level, thereby disrupting RNA metabolism and normal cellular functions (Huang and Chen, 2023). Consequently, METTL3 and ZC3H13 present as potential therapeutic targets for the treatment of adenomyosis. In addition, the dysregulated expression of LASP1 in adenomyosis is closely associated with the DNA methylation status in the promoter region of the LASP1 gene. Meanwhile, studies have confirmed that the overexpression of LASP1 can promote the invasion and proliferation of ectopic lesions (Liu et al., 2021a). Therefore, LASP1 may be a promising therapeutic target for adenomyosis.

In addition to methylation, aberrant histone modifications also offer therapeutic avenues. For instance, trichostatin A (TSA), a histone deacetylase (HDAC) inhibitor, has been demonstrated in studies to suppress the activity and cell cycle progression of ectopic endometrial stromal cells by regulating epigenetic modifications (Jichan et al., 2010). Furthermore, lysine demethylase 1A (KDM1A) is highly expressed in adenomyosis; silencing KDM1A can inhibit cell proliferation, migration, and invasion (Cui et al., 2024), indicating its potential as a therapeutic target for adenomyosis.

### 4.3 Natural products

Natural compounds have substantial therapeutic potential because of their impact on several genes and pathways, whether they are utilized as adjuvant medicines or in conjunction with traditional therapy regimens. The majority of natural products act as modulators of the signaling pathways they are linked with, as opposed to direct inhibitors or activators that target particular signaling pathways. That is to say, without interfering with homeostasis or the regular growth and development of normal tissues, these natural compounds can preserve or restore the balance of relevant signaling pathways during prevention or treatment.

Numerous studies have indicated that some dietary polyphenols play a significant role in cancer treatment by inducing apoptosis, modulating immune responses, reducing angiogenesis, inhibiting cell growth, and inactivating carcinogens (Anjum et al., 2022). *In vitro* studies on human ectopic endometrial stromal cells and animal experiments, it has been shown that natural plant active compounds such as parthenolide and andrographolide can inhibit the secretion of inflammatory cytokines and reduce lesion proliferation by suppressing the NF-κB signaling pathway (Meresman et al., 2021); curcumin and ginsenosides can alleviate inflammatory responses by inhibiting the NF-κB signaling pathway to reduce the expression of pro-inflammatory factors, and can also decrease angiogenesis by downregulating the VEGF signaling pathway (Meresman et al., 2021); resveratrol can reduce the proliferation of endometrial tissue by inhibiting the ER-α. Furthermore, studies on mouse models have found that resveratrol treatment can alleviate endometrial fibrosis and regulate the expression of immune regulation-related genes, thereby slowing down the progression of adenomyosis (Zhu et al., 2023); epigallocatechin-3-gallate (EGCG) can inhibit angiogenesis and VEGF expression, induce apoptosis, and reduce fibrosis (Alam et al., 2024). Additionally, research on adenomyosis mouse models has indicated that EGCG can inhibit the infiltration of the uterine myometrium, decrease plasma corticosterone levels, and reduce uterine contractility. Meanwhile, it has also been found that EGCG can alleviate hyperalgesia in adenomyosis by increasing inhibitory gamma-aminobutyric acid (GABA)-ergic neurons. Therefore, EGCG holds promising therapeutic potential in adenomyosis, but further research is still needed (Hazimeh et al., 2023); P. polyphylla and Cassia twig can induce apoptosis of ectopic endometrial cells by inhibiting the PI3K/AKT pathway, thereby slowing down the progression of adenomyosis (Shi et al., 2024).

Based on the above, it is evident that natural products hold significant potential for targeting signaling pathways associated with adenomyosis. Compared to conventional pharmaceuticals, natural product drugs generally exhibit lower toxicity and fewer side effects. Additionally, natural products also have relatively low costs. However, several challenges remain in the development of natural product therapeutics, including standardization of plant extraction and patenting issues that require urgent resolution. Moreover, due to the heterogeneity and relative instability of natural products, their pharmacokinetics and pharmacodynamics have often not been thoroughly investigated (Meresman et al., 2021).

## 5 Conclusion

With the increasing number of studies on adenomyosis, we can realize the important role of signaling pathways in adenomyosis. The therapy of adenomyosis is hampered by the disease’s currently unknown etiology and pathophysiology as well as the intricacy of associated signaling networks. The current research on these medications has already given us hope for more precise treatment for adenomyosis (Table 1), which will also become increasingly important for adenomyosis patients with fertility needs in the future, even though there is still much work to be done in research targeting these signaling pathways and more thorough studies are required for their wide-spread clinical application. In the future, more attention should be paid to studies related to targeted signaling pathways. In addition, emerging research directions such as multi-target combination therapy, epigenetic regulation, and research on natural products cannot be ignored.

**TABLE 1:** Targeted therapy and mechanisms of signaling pathways related to adenomyosis.

	Treatment	Pathway	Mechanism	References
Progestogen and Estrogen	LNG-IUS	Progestogen	PR	Kho et al. (2021)
	Mifepristone Ulipristal		PR	Che et al. (2020), Yoon and Yuk (2021)
	EX-527		SIRT1	Marquardt et al. (2023)
	GnRH	Estrogen	HPO axis	Etrusco et al. (2023)
	Aromatase inhibitors		Aromatase	Liu et al. (2021b)
Proliferation and invasion	LGK974	WNT	PORCN	Liu et al. (2022), Maurice and Angers (2025)
	XAV939		TNKS	Maurice and Angers (2025)
	scFv–DKK1c		Wnt substitute molecule	
	SFRP2		β-catenin	Yildiz et al. (2023)
	PROTACs	PI3K/AKT	Degrade the target protein	Kelly et al. (2024)
	DUSP4	MAPK	Dephosphorylation	Mandal et al. (2023)
	HAND2-AS1		FGFR	Xiang et al. (2019)
	Qiu’s Neiyi Recipe		MAPK/ERK	Ying et al. (2021)
Inflammation and fibrosis	Aspirin Sulfasalazine	NF-κB	IKK	Guo et al. (2024)
	Bortezomib Carfilzomib		Proteasome	
	NKILA		IκBα	Ahmad et al. (2022)
	cGAs catalytic site inhibitors DNA binding blockers	cGAS-STING	cGAS	Zhang et al. (2023)
	CDN binding antagonists STING palmitoylation inhibitors		STING	
	Pirfenidone	TGF-β	TGF-β	Yoo et al. (2020)
	circRNA		TGF-β/Smad	Zheng et al. (2022)
	CircEIF3I		SMAD 3	Zhao et al. (2023)
Antiangiogenesis	Axitinib	VEGF	VEGF-R2 tyrosine kinase	Harmsen et al. (2025)
Emerging	Vismodegib Sonidegib Glasdegib	Hedgehog	SMO	Zhang and Beachy (2023)
	Fab^6^ᴴ^3^		PTCH1	Hu et al. (2025)
	let-7a	Hippo	Hippo-YAP1	Huang et al. (2021)
	Verteporfin		YAP	Jin et al. (2023), Lin et al. (2023)
Multitarget	ARG2	NF-κB、WNT		Xu et al. (2024)
	Melatonin	NF-κB、VEGF		Hung et al. (2021)
	Imperatorin	NF-κB、PI3K/AKT		Ma et al. (2021)
